# The spatio-temporal evolution of leishmaniasis in the province of Essaouira

**DOI:** 10.3389/fepid.2024.1462271

**Published:** 2024-12-02

**Authors:** Said Benkhira, Najma Boudebouch, Bouchra Benazzouz

**Affiliations:** ^1^ Laboratory of Biology and Health, Ibn Tofail University, Kénitra, Morocco; ^2^ Higher Institute of Nursing and Technical Health Professions, Ministry of Health and Social Protection, Marrakech, Morocco

**Keywords:** cutaneous leishmaniasis, spatio-temporal evolution, parasitic diseases, *Leishmania tropica*, Essaouira province

## Abstract

**Introduction:**

Leishmaniasis is a highly prevalent neglected tropical disease. It represents a significant public health concern in northern Africa, particularly in Morocco. To assess the extent of the disease at the provincial level, as well as the temporal evolution of CL cases and their geographic distribution.

**Methods:**

834 cases of cutaneous leishmaniasis (CL) diagnosed positive by the hygiene and health laboratory of the health delegation of the province of Essaouira during the period from January 1st, 2014 to December 31st, 2023.

**Results and discussion:**

Among the 57 communes of the province, three are hyper-endemic and represent the main foci of LC; Elhanchan, Had Draa, Smimou with 66.42% of cases. Other communes with significant increases include Aguerd, with 15.6% of cases, an incidence peak of 279.7 per 100,00.0 in 2022, and Bizdad, 11.8% with an average incidence of 41.1 per 100,000. The transmission of the parasitosis continues to spread to create new outbreaks each year and reach 25 municipalities in the province which have experienced at least one positive case in 2023. Two new outbreaks appeared after 2018 in Sidi Kaouki (5% of cases) and Tidzi (5.6%). The temporal analysis shows a significant rise in cases over time, with an annual average of 83 cases. The trend paused during the COVID-19 lockdown but resumed exponentially, peaking in 2023. The overall incidence in the province increased from 11.1 per 100,000 in 2015 to 40.3 per 100,000 in 2023, with a significant rise over the study period (*p* < 0.001). The average incidence during this time was 18.32 per 100,000, showing considerable variability across different years.

**Conclusion:**

The spread of cutaneous leishmaniasis in the province of Essaouira is multifactorial and results from the complex interaction between vectors, parasites, the environment, and human behaviors. A better understanding of these factors is essential to developing effective disease prevention and controlling strategies.

## 1 Introduction

Leishmaniasis is a group of parasitic diseases caused by protozoan flagellates belonging to the genus *Leishmania* (1, 2) that require a female phlebotomine sand fly for transmission (3, 4) and is considered the third most important vector-borne disease after malaria and filariasis (5). *Leishmania* parasites infect macrophages (6), leading to three primary forms of leishmaniasis: cutaneous, the most common; mucocutaneous; and visceral, which is the most severe (2, 7). Leishmaniasis is endemic in 99 countries across all inhabited continents except Oceania (8). The latest estimation indicates annually 600,000 to 1 million cutaneous leishmaniasis (CL) cases and 50,000 to 90,000 visceral leishmaniasis (VL) cases (9).

In North Africa, CL is highly prevalent and represents a serious public health problem, especially in Morocco, Algeria, Tunisia, and Libya; however, the case burden is lower in Egypt (10). This emerging disease is closely linked to environmental complexity and is reflected in the diversity of leishmaniasis (11). According to the WHO, Morocco is characterized by several vegetation zones that reflect a varied climate and topography and influence the distribution and density of sandfly (12).

The epidemiology of cutaneous leishmaniasis in Morocco is complex and less well understood than that of either visceral leishmaniasis. Among the three clinically important *Leishmania* species (*L. major, L. tropica, L. infantum*), *L. tropica* has the largest geographic distribution and is considered a public health threat by the Moroccan Ministry of Health reference (13). The disease occurs at hypoendemic intensity in separate foci between Tadla and Agadir, in the 'subhumid' climate zone north and west of the High Atlas (13). A large focus, spanning an area of approximately 400 km^2^ from Azilal in the center to Essaouira in the west and Agadir-Guelmim in the south, has been identified as a hotspot for cutaneous leishmaniasis caused by *L. tropica* in our country (14, 15).

*Leishmania* infections caused by *L. tropica* continue to emerge. Recent studies have reported a new focus of anthroponotic cutaneous leishmaniasis (16, 17). In Morocco, CL due to *L. tropica* has risen since the 1980s and has spread widely to become the most abundant form of leishmaniasis in the territory (18). Rural and semi-urban sectors of the province of Essaouira present, more than others, favorable conditions for the proliferation of the vector and the parasite reference. The anthroponotic transmission is so far the only recognized mode, despite recordings of *L. tropica* infection in animal hosts in other countries (13). The epidemic status of CL caused by *L. tropica* in Morocco and the increased movement of the population from rural to urban areas indicates a possible introduction of this species to urban areas (19).

In the latest studies, the spatial distribution of CL in Essaouira Province showed that the districts with the highest CL case burden were located in the center and southwestern areas of the province: Districts Had Draa, Smimou and Elhanchan while the lowest burden was found in districts in northwestern areas (10).

An earlier retrospective clinicoepidemiological study carried out covering the last decade in Essaouira provinces shows a significant increase in the number of cases since 2021 (Benkhira and al unpublish data). On the other hand, several cases of leishmaniasis were reported near the urban municipality of Essaouira and highlighted the possible introduction of *L. tropica* to urban areas which raises the alarm as well as the emergence of new outbreaks, which represents a challenge for health officials and professionals and calls for strengthening the surveillance system by organizing active mass screenings, improving diagnostic means, and raising awareness among the population about protective measures and hygiene against CL.

In this context, we have undertaken this study whose main objective is the study of the spatial evolution of CL incidence due to *Leishmania tropica* in different municipalities in the province of Essaouira and especially the appearance of new endemic foci around urban areas during the period from 2014 to 2023.

## 2 Materials and methods

### 2.1 Study area

#### 2.1.1 Geographic situation

**Figure 1:**
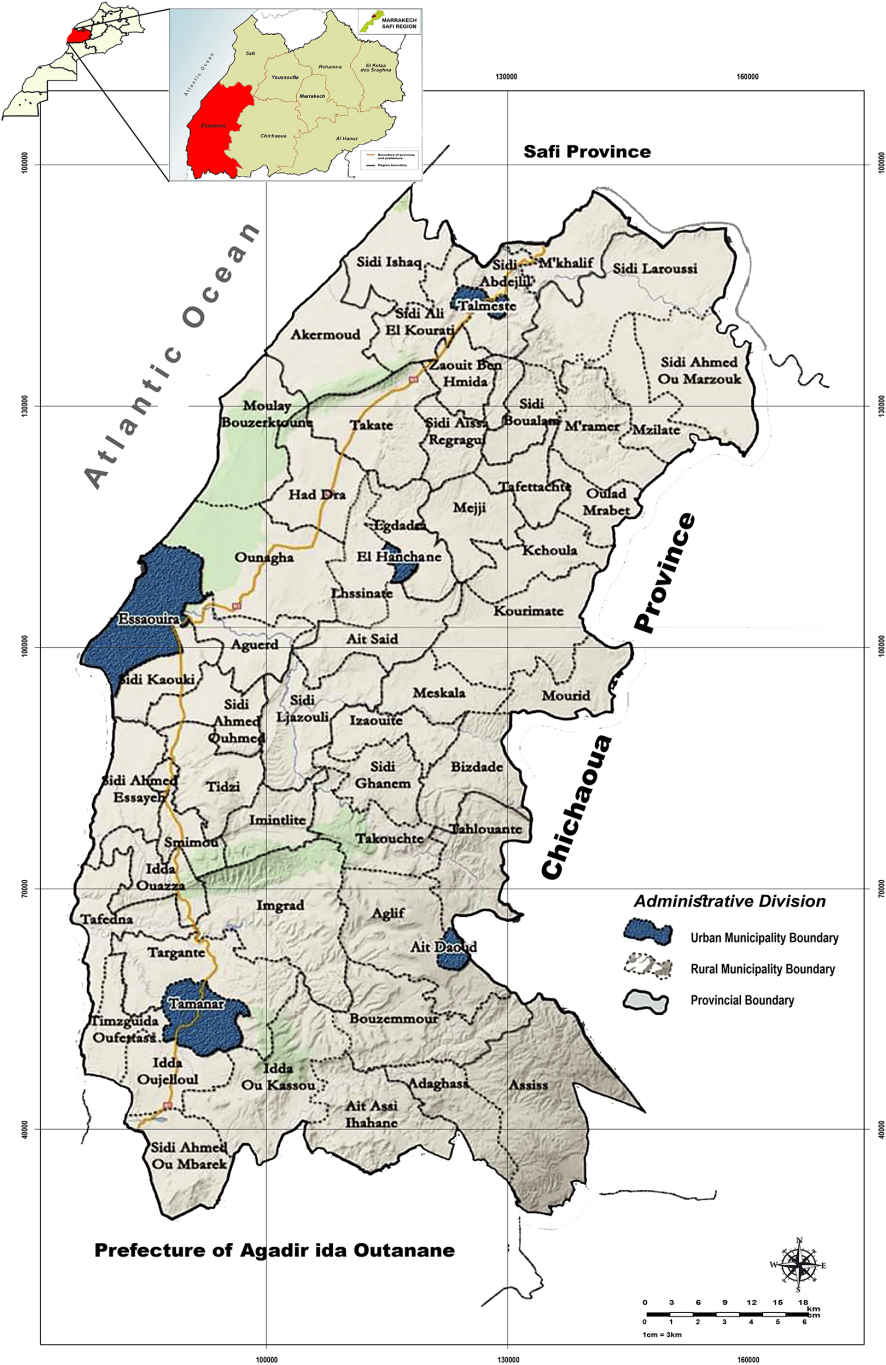
Location and topographical map of the province of Essaouira.

Essaouira province is part of the region of Marrakech-Safi (Morocco). It is geographically bounded by the Safi province to the north, the prefecture of Agadir to the south, the Chichaoua province to the east, and the Atlantic Ocean to the west (20) (Figure 1). It encompasses a total area of 6,355 square kilometers (21). The province of Essaouira is characterized by a semi-arid climate, which remains generally hot and dry throughout the year (10, 22).

#### 2.1.2 Demographic data

The population is estimated at 446,979 inhabitants, according to the 2014 national census. Rural residents make up 77.6% of the population, accounting for 337,672 people, while 22.4% live in urban areas, totaling 109,307 inhabitants. Young people form the largest segment of the population (23). The population of the province experiences higher poverty levels compared to the rest of the region. The local economy primarily relies on agriculture, livestock, tourism, and fishing. Livestock farming, which supplements agricultural activities, serves as a crucial source of income for the rural population, representing over 76% of the inhabitants (10).

#### 2.1.3 Subdivision territorial

The province is composed of 57 administrative communes, 5 municipalities and 52 rural communes (20) (Figure 1). These communes are grouped into 11 health constituencies (sectors) according to the 2014 health division.

### 2.2 Data source

We conducted a retrospective study, which allowed us to collect 834 cases of cutaneous leishmaniasis (CL) due to *L. tropica* diagnosed as positive by the hygiene and health laboratory of the health delegation of the province of Essaouira, during the period from January 1st, 2014, to December 31st, 2023. The data were collected as part of the Parasitic Disease Control Program, which is integrated into the Integrated Vector Management (GILAV) program adopted by the Ministry of Health since 2005. The objective of this program is the surveillance and control of vector-borne diseases (24). The data collected are the numbers of cases reported annually, based on the area of origin of each case. The demographic data for the population of the various municipalities within the province were gathered by the statistical unit of the provincial delegation of health.

### 2.3 Method of analysis

To evaluate the extent of the disease at the provincial level, along with the temporal trends and geographical distribution of LC cases due to *L. tropica*, data are analyzed according to the incidence of cases during the study period. This incidence is calculated annually per 100,000 inhabitants for each constituency by subtracting the LC of the province, as shown in (Table 1). Demographic data are presented as proportions or averages values. Linear regression was used to estimate differences in annual incidence (dependent variable) over the study period (from 2014 to 2023). For each district, we used monthly case data over several years and applied an independent Chi-square test. This test assessed whether the monthly distribution of cases in each district differed significantly from a uniform distribution. Here, a *p*-value less than 0.05 indicated that leishmaniasis cases were distributed non-uniformly throughout the year, suggesting a seasonal trend within the commune.

**Table 1:** Evolution of LC in the health constituencies of Essaouira province from 2014 to 2023.

	2014	2015	2016	2017	2018	2019	2020	2021	2022	2023	Total	Average
Elhanchane	16	13	22	23	18	23	16	20	21	84	256	25,6
Population	47,071	46,058	46,058	45,434	45,222	45,002	43,232	47,161	47,140	47,109		
Incidence^a^	34	28	28	51	40	51	37	42	45	178		53
Had Dra	28	25	16	18	15	16	4	13	14	36	185	18,5
Population	38,807	37,837	37,837	37,122	36,882	36,635	38,642	38,584	33,484	38,446		
Incidence^a^	72,1	66,0	42,0	48,0	40,6	41,0	10,0	33,6	42,0	93,6		49
Smimou	11	9	18	13	20	18	9	11	7	17	133	13,3
Population	44,664	43,547	43,292	42,724	42,448	42,155	44,474	38,946	44,331	44,248		
Incidence^a^	24,6	20,6	41,5	30,4	47,1	42,6	20,2	28,2	15,7	38,4		41,1
Bizdad	3	0	7	5	11	9	12	11	20	21	99	9,9
Population	24,087	23,485	23,347	23,041	22,892	22,739	23,984	23,948	23,907	23,863		
Incidence^a^	12,4	0,00	30,0	21,7	48,0	39,5	50,0	37,5	83,6	88,0		
Aguerd	0	2	6	12	8	9	12	15	42	25	131	13,3
Population	13,027	12,701	12,627	12,462	12,381	12,299	12,972	12,952	15,012	12,906		
Incidence^a^	0,00	15,7	47,5	96,2	56,5	56,9	53,9	84,9	279,7	193,7		88,5
Tamanar	5	1	0	0	0	4	0	1	0	0	11	1,1
Population	52,108	51,152	51,160	50,705	50,550	50,382	47,416	52,572	52,606	52,629		
Incidence^a^	11,5	01,9	0,00	0,00	0,00	07,9	0,00	01,9	0,00	0,00		2,3
Talmest	1	0	0	1	0	0	1	0	0	0	3	0,3
Population	52,380	51,212	51,038	50,457	50,202	49,937	52,418	52,391	52,353	52,304		
Incidence^a^	01,9	0	0	02	0	0	02	0	0	0		0,6
Tafetacht	0	0	0	0	0	0	1	0	0	0	1	0,1
Population	46,675	45,508	45,241	44,648	44,360	44,062	46,476	46,406	46,328	46,241		
Incidence^a^	0	0	0	0	0	0	02,1	0	0	0		0
Essaouira	0	0	0	3	1	3	1	2	1	3	14	1,4
Population	81,384	81,791	83,488	83,913	84,591	85,237	85,551	86,309	87,033	87,722		
Incidence^a^	0	0	0	03,5	01,2	03,5	01,1	02,3	01,1	03,4		1,6
Province	64	50	69	75	73	82	56	73	105	187	834	83,4
Population	459,008	450,713	450,903	446,979	445,673	444,273	462,931	463,389	463,738	463,979		
Incidence^a^	13,9	11,1	15,3	16,6	16,3	18,4	12	15,7	22,6	40,3		18,22

^a^ The annual incidence rate of CL in each commune was calculated using the following formula: incidence rate = (total number of CL cases per year/total population at risk) × 100,000.

Districts with multiple months of zero values were excluded from this analysis, as these could skew statistical results by introducing artificial variability.

At the provincial level, we aggregated the monthly case totals from all communes included in the study. We then applied a Chi-square test on this aggregated dataset to evaluate whether the distribution of cases across months deviated from a uniform distribution. A *p*-value below 0.05 at the provincial level indicated that case distribution was not random across months, suggesting possible seasonal trends or the influence of environmental and climatic factors throughout the province.

The results and data are processed using Excel software 2016 and Rstudio software 4.4.1.

The procedures followed complied with the ethical standards of the competent commission for human experimentation and with the principles of the Declaration of Helsinki. Authorization was obtained from the provincial delegation of the Ministry of Public Health.

## 3 Results

### 3.1 Geographical distribution of cutaneous leishmaniasis cases throughout the province

**Figure 2:**
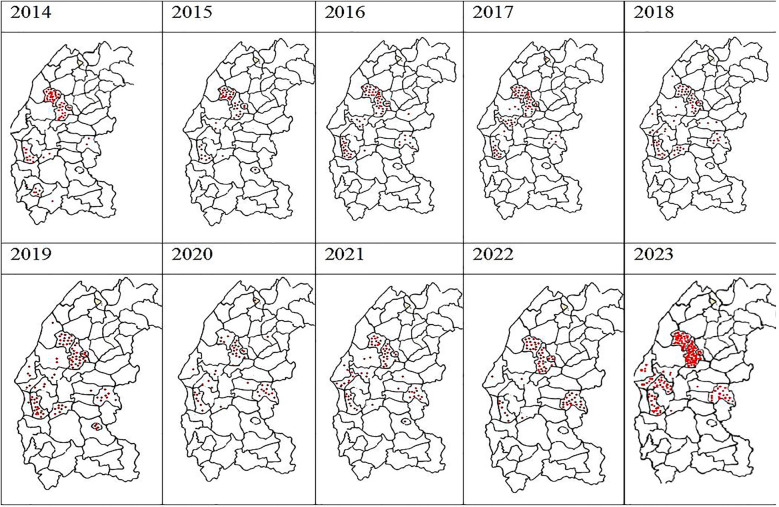
Spatiotemporal heterogeneity of the number of cases of autochthonous human leishmaniasis in Essaouira province from 2014 to 2023 per municipality.

**Figure 3:**
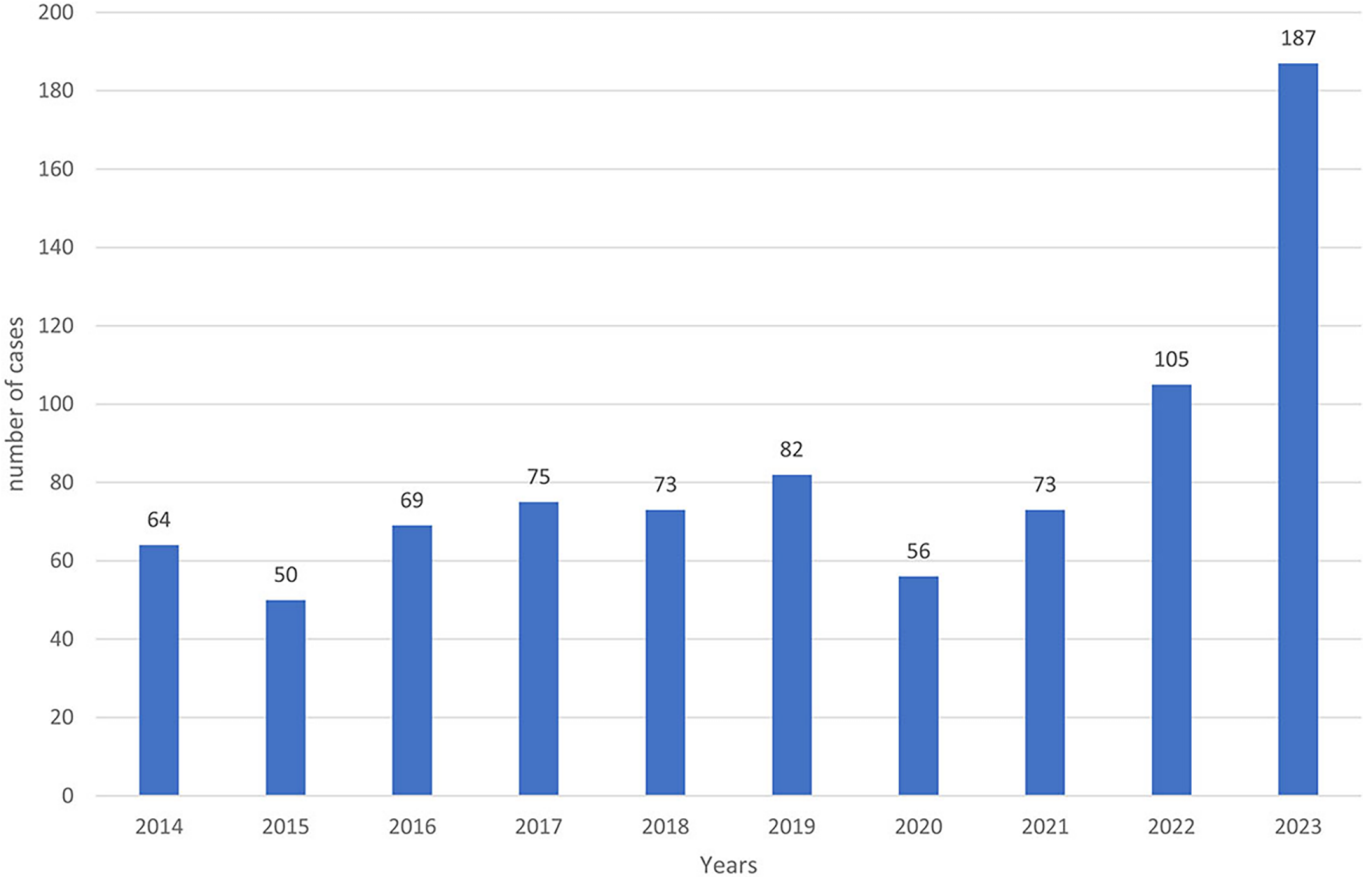
Evolution of the number of LC cases over years.

From 2014 to 2023, a total of 834 positive cases of cutaneous leishmaniasis (CL) were diagnosed in the province of Essaouira. The analysis of the geographical distribution reveals significant disparities across the province's municipalities and communes; out of the 57 communes, three are hyper-endemic and account for the majority of CL outbreaks: Elhanchan, Had Draa, and Smimou, together representing 66.42% of all cases (Table 2). Over the years, the number of affected municipalities and communes increased from 7 (12%) in 2014 to 25 (44%) in 2023 (Table 1), (Figure 2). The highest number of cases occurred in 2023, reaching 187 cases (22%), while the lowest was recorded in 2015, with 50 cases (6%) (Figure 3).

**Table 2:** Number of LC cases recorded in each municipality and rural commune over time during the last decade.

	2014	2015	2016	2017	2018	2019	2020	2021	2022	2023	Total
Elhanchane	16	13	22	23	18	23	16	20	21	84	256
Had Dra	28	25	16	18	14	14	4	11	14	36	180
Smimou	9	8	17	13	13	18	8	10	7	17	120
Bizdad	2	0	7	3	3	8	11	4	12	18	68
Sidi kaouki	0	0	0	0	3	2	2	8	21	6	42
Aguerd	0	2	6	11	1	2	2	3	1	2	30
Imintlit	2	1	1	0	6	0	1	1	0	0	12
Aid Daoud	0	1	0	0	0	4	0	1	0	0	6
Ounagha	0	0	0	0	1	2	0	2	0	0	5
Ait Bayoud	0	0	0	1	3	0	0	0	0	0	4
Ida Ouzemzem	0	0	0	0	2	0	0	4	8	2	16
Meskala	0	0	0	0	0	0	0	1	0	0	1
Tamanar	5	0	0	0	0	0	0	0	0	0	5
Ait Zelten	1	0	0	0	0	0	0	2	0	0	3
Ida Ou Kazzou	1	0	0	0	0	0	0	0	0	0	1
Kourimate	0	0	1	0	0	0	0	0	0	0	1
Sidi Hmad ou Hamed	0	0	0	1	3	3	3	0	2	0	12
Talmest	0	0	0	1	0	0	0	0	0	0	1
Ezzaouite	0	0	0	1	3	1	1	1	0	1	8
SAS	0	0	0	0	1	0	0	0	0	0	1
Tafetacht	0	0	0	0	0	0	1	0	0	0	1
Barakat Radi	0	0	0	0	0	0	1	0	0	0	1
TIDZI	0	0	0	0	1	2	5	4	18	17	47
Essaouira	0	0	0	3	1	2	1	2	1	4	14
Ghazoua	0	0	0	0	0	1	0	0	0	0	1
Total	64	50	69	75	73	82	56	73	105	187	834

**Figure 4:**
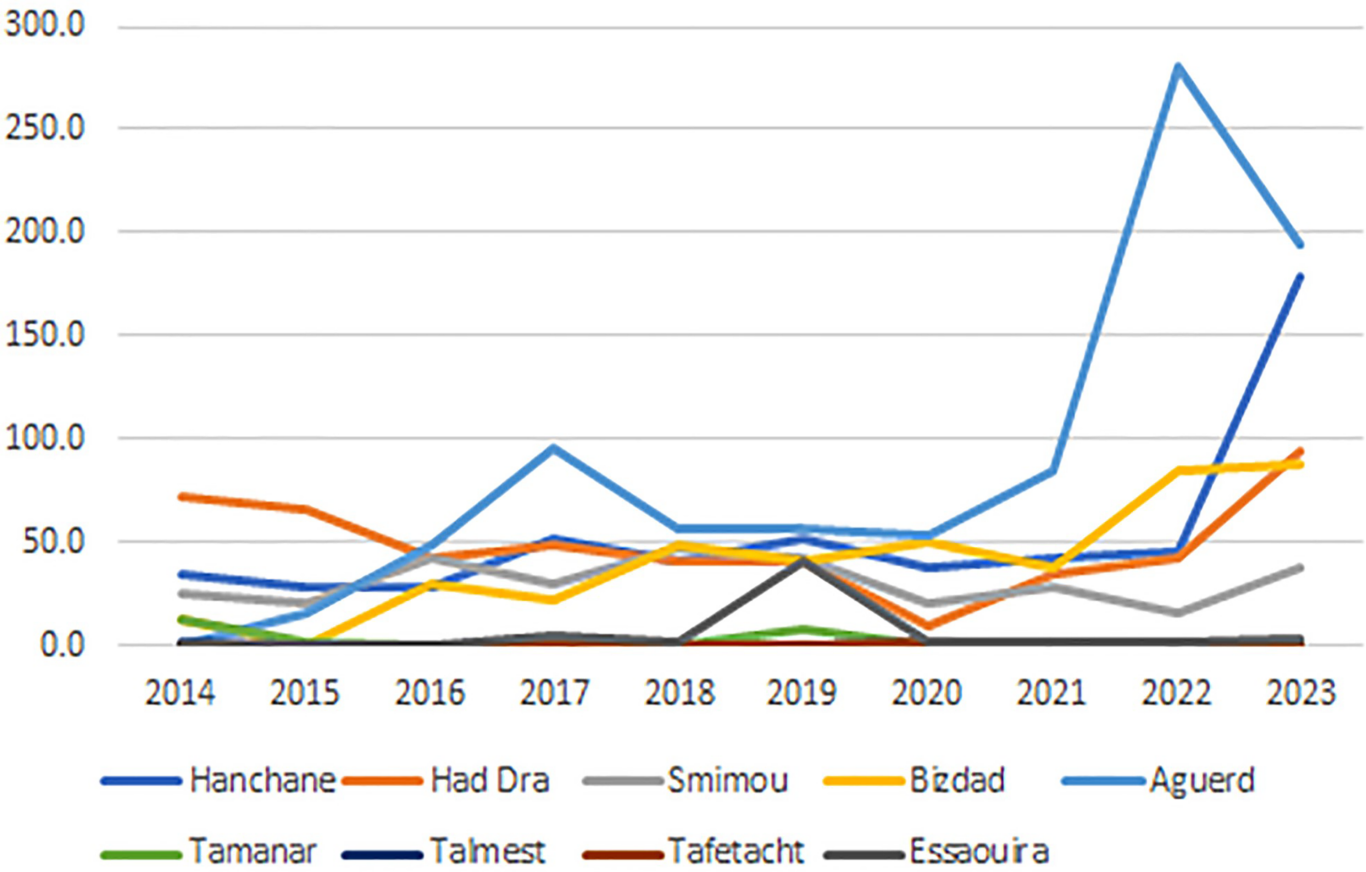
The cumulative incidence of LC in the different constituencies of the province from 2014 to 2023.

Elhanchan has the highest proportion of cases, accounting for 30.45% (256 cases) of the total. Notably, 49% of these cases have been registered since 2021. The incidence in Elhanchan increased significantly, rising from 28 per 100,000 inhabitants in 2015 to 178 per 100,000 in 2023, the average is 53/100,000. Had Draa is the second constituencies with 22% (185 cases) of cases with an incidence average of 48.9/100,000, followed by Smimou 15.9% (133 cases) and an incidence average of 30.9/100,000 (Table 2) and (Figure 4).

Additionally, two other constituencies exhibit endemic characteristics: Aguerd, accounting for 15.6% of cases (131 cases), showed a significant increase in incidence, from 0 in 2014 to 279.7 per 100,000 in 2022, with an average incidence of 88.5 per 100,000, the highest among all municipalities in the province. Bizdad, representing 11.8% of cases (99 cases), reported an incidence of 41.1 per 100,000 (Table 2) and (Figure 4).

Furthermore, from 2018 two new outbreaks appeared; the first in Sidi Kaouki 5% (42 cases) and the second in Tidzi 5.6% (47 cases) (Table 2).

Eight other municipalities recorded sporadic cases, with each reporting just 1 case during the whole period of the study.

The main results of the spatio-temporal distribution of CL in the province of Essaouira reported during the last decade are summarized in Figure 2.

### 3.2 Temporal distribution of CL cases from 2016 to 2023

The temporal analysis highlights a significant increase in the number of cases over time, with an annual average of 83 cases. This trend was briefly interrupted by the COVID-19 lockdown in 2019 and 2020 but saw exponential evolution since 2021, with a maximum of 187 cases recorded in 2023 (Figure 3).

**Figure 5:**
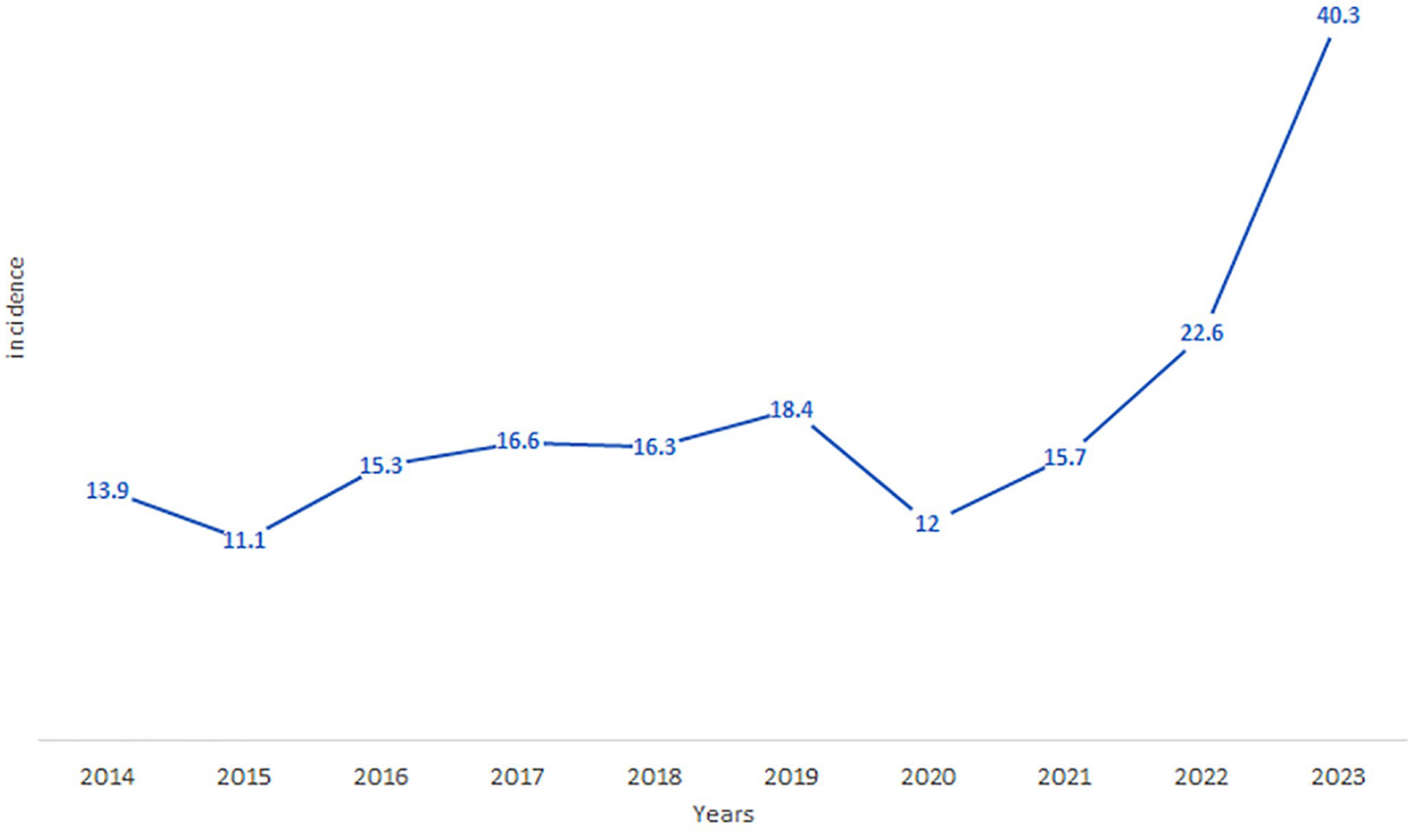
The evolution of the cumulative incidence of LC in the province of Essaouira from 2014 to 2023.

The province-wide annual cumulative incidence also displayed an exponential rise over the study period, increasing from 11.1 per 100,000 inhabitants in 2015 to 40.3 per 100,000 in 2023 (*X*-squared = 34.489, df = 9, *p*-value = 7.331e-05, *p* < 0.001) (Figure 5). The average incidence over the study period was 18.32 per 100,000 inhabitants (SD = 8.38), and the range difference of 29.2 per 100,000 reflects considerable variability in incidence rates.

The monthly distribution of leishmaniasis cases exhibits significant variation across health districts. In particular, Elhanchan (*p* = 6.01e-10), Aguerd (*p* = 6.32e-16), Bizdad (*p* = 1.78e-2), and Smimou (*p* = 5.26e-4) show *p*-values below the 0.05 significance threshold, indicating a statistically significant difference in case distribution across months. This pattern suggests that cases are not uniformly distributed throughout the year in these districts. In contrast, Had Draa (*p* = 7.49e-2) show *p*-values consistent with a uniform distribution, indicating no significant monthly variation. To improve the relevance and robustness of our *χ*^2^ test, we decided to exclude certain districts with null values for several months (Tmanar, Talmest, and Essaouira).

At the provincial level, the analysis reveals a highly significant difference in case distribution across months (*χ*^2^ = 169.82, df = 11, *p*-value < 2.2e-16). Certain months, such as March and April, have higher-than-average case counts, while August, September, and November exhibit lower counts. This temporal variation suggests the presence of seasonal patterns or other factors influencing the incidence of cases throughout the year (Table 3).

**Table 3:** The distribution of leishmaniasis cases by month in the districts and in the province of essaouira.

Location	Jan	Feb	Mar	Apr	May	Jun	Jul	Aug	Sept	Oct	Nov	Dec	Total cases	Chi-squared (*X*^2^)	*p*-value
Aguerd	13	6	37	19	11	8	6	6	2	5	4	7	145	97.2	6.32E-16
Bizdad	11	15	12	17	9	11	6	7	5	6	4	4	109	23	0.0178
Had draa	22	16	22	22	23	13	11	10	12	12	14	11	166	18.3	0.0749
Hanchan	19	32	45	37	23	23	17	9	9	18	12	13	254	66.3	6.01E-10
Smimou	12	9	13	20	17	17	9	6	4	7	3	6	113	33	0.000526
Province	81	82	135	117	87	74	52	39	34	51	39	44	834	166.43	<2.2e-16

## 4 Discussion

The present findings showed the significant increase in the incidence of leishmaniasis in the province of Essaouira during the last decade, with the appearance of new outbreaks of leishmaniasis. These outbreaks are located at the borders of the city of Essaouira and therefore the possible introduction of leishmaniasis in the urban perimeter of the city of Essaouira. Indeed, the latest study conducted Between 2000 and 2016, in Essaouira Province showed an annual incidence rate, which fluctuates between 3.5 and 19.9 cases per 100,000 inhabitants, with an average of 10.6 cases/year (10). In contrast, our results show an increasing from 11.1 per 100,000 inhabitants in 2015 to 40.3 per 100,000 in 2023, with an average incidence of 18.32 per 100,000 inhabitants. We report a change in the distribution of CL with more CL cases being seen in many new rural and peri-urban districts in Essaouira.

In Morocco, CL due to *L. tropica* has risen since the 1980s and has spread widely to become the most abundant form of leishmaniasis in the territory. Since 1990s, several epidemics with hundreds of cases have been reported in several Moroccan cities (15), possibly as a result of migrations and population movements from endemic to non-endemic cities as well as human population growth in poor habitat (19). In fact, the emergence or reemergence of the disease is under the control of several factors, mainly environmental, which are crucial in initiating the spread of the parasite among the human population, socioeconomic factors, which potentially impact the morbidity (18), and the climate changes can impact the extension of geographical distribution of *Phlebotomus Sergenti*, which is the proven vector of *L. tropica* in Morocco, resulting in the spread of anthroponotic CL in both rural and urban/peri-urban areas (19).

*Phlebotomus sergenti* is abundantly present all around the mediterranean basin. Its transmission cycle is still subject to controverse. In some countries, the presence of an animal reservoir has been confirmed. However, the anthroponotic transmission is so far the only recognized mode, despite recordings of *L. tropica* infection in animal hosts (18).

According to data from the provincial health delegation of Essaouira, the first case of cutaneous leishmaniasis due to *L. tropica* was recorded in 1988 in the province of Essaouira in Smimou district. In 1990, an investigation revealed 21 cases on the same site, before recording an epidemic outbreak in Had Draa and Elhanchane in 2006 (43 cases) (unpublish data). Since then, the transmission has continued to spread, creating new outbreaks each year and reaching 25 municipalities with at least one positive case during 2023. In 2020, we noted the reduction in screening, prevention and control activities of the National Program for Surveillance and Control of Leishmaniasis (Programme national de lutte contre les leishmanioses PNLL), the leishmaniasis during the covid-19 crisis (25). A comparison of case distribution across years with other provinces showed that, between 2016 and 2021, the provinces of Taourirt, Nador, Jrada, and Figuig recorded the highest values in 2018, while the province of Essaouira reported its peak case numbers in 2019 during the same period (26).

The geographical common point between the epidemic focis is their positioning in the center of the province. The study by Elaasri et al. in 2016 associates spatial distribution with the action of environmental factors, especially those related to climate and geography thus low-altitude areas exposed to oceanic effects experience fewer cases than high and continental areas (27). Other studies associate the geographical distribution with the action of intrinsic factors of the parasite and its cycle which are also linked to environmental factors (28). In endemic areas, increased risk of leishmaniasis is associated with poverty, poor housing and poor sanitation, which lead to the proliferation of vectors and their access to humans (28). The Essaouira province is one of the poorest provinces in the kingdom and is composed mostly of a rural population (29).

In our study, 40% of cases were recorded in the spring (March to May), a finding similar to that of Fatima et al., whose study on the distribution of *L. tropica* in the Settat region found that the majority of cases occurred between February and May (30). In contrast, studies by Saadia Achichaou et al. (2012–2022 in the Errachidia province) and Ibrahim Mouloudi et al. (2016–2021 in the eastern region of Morocco) reported a seasonal concentration of leishmaniasis cases in the autumn-winter months (26, 31). Furthermore, another study by Mouloudi in the Jerada area showed that cutaneous leishmaniasis cases peak cyclically in December and January (32).

This variation in seasonal patterns can likely be attributed to the dominant type of *Leishmania* in each region; The Errachidia and eastern regions, are known for endemic areas for *Leishmania major* (5, 33). In Morocco, the epidemiology of cutaneous leishmaniasis due to *L. tropica* remains partially understood (34). The seasonal variations observed may be attributed to the presence and activity patterns of vectors and reservoirs, which are strongly influenced by climatic conditions. Seasonal trends in leishmaniasis cases likely reflect the life cycle of the vectors and the incubation period of the disease, with symptoms (such as skin lesions) usually appearing one to two months or more after an infectious bite from female sandflies (26).

The current epidemiological situation of leishmaniasis in the Kingdom, which has become worrying due to increasing morbidity and the continuous identification of new active transmission foci (35). On the other hand, the reactivation of existing foci in peri-urban and rural areas persists due to the persistence of risk factors (36). These factors may be related to Human activities, such as deforestation, unplanned urbanization, and human migration, can disrupt the natural habitats of sandflies and environmental factors, thus promoting the spread of leishmaniasis (37). The proximity to natural vegetation remnants increased disease risk (11).

These present findings confirm the insufficiency of the measures deployed through the leishmaniasis monitoring program. The CL control policy recommends potential interventions to reduce the transmission of *L. tropica,* include screening and treatment of infected patients, vector control measures and improvement of hygiene conditions (38). Therefore, there is a real need for the implementation of novel prevention strategies, as well as improved health education by informing people and physicians about CL in both endemic and non-endemic regions.

## 5 Conclusion

Despite the efforts made by the public authorities, cutaneous leishmaniasis continues to pose a significant public health challenge in Morocco. The current situation in the Essaouira province shows a notable increase in incidence, as well as an expansion in the distribution of cases in peri-urban areas particularly the appearance of new outbreak around the Essaouira city especially in the constituencies of Aguerd.

The control of cutaneous leishmaniasis in the province of Essaouira requires an integrated approach that combines epidemiological surveillance, vector control and awareness raising among local populations. Prevention must include hygiene education, rigorous waste management to limit vector habitats, and access to early treatment. Concerted action between health authorities, researchers and local communities is crucial to controlling the spread of the disease, especially in endemic areas.

Further studies are needed to better understand the epidemiology of the disease in the region.

### 5.1 The limits of the study

The main limitations of this study are:

- Lack of information on patient movement in endemic areas.
- The distinctions between urban and rural areas are strictly administrative and do not reflect the predominantly rural nature of the majority of the province.

## Data Availability

The original contributions presented in the study are included in the article/Supplementary Material, further inquiries can be directed to the corresponding author.
